# Targeting Bacterial Infections in Periodontal Disease: From Conventional Antibiotics to Next-Generation Therapeutics

**DOI:** 10.3390/antibiotics15040397

**Published:** 2026-04-14

**Authors:** Nada Tawfig Hashim, Rasha Babiker, Muhammed Mustahsen Rahman, Riham Mohammed, Vivek Padmanabhan, Md Sofiqul Islam, Mariam Elsheikh, Salma Musa Adam Abduljalil, Ghiath Mahmoud, Nallan C. S. K. Chaitanya, Bogahawatte Samarakoon Mudiyanselage Samadarani Siriwardena, Ayman Ahmed, Bakri Gobara Gismalla

**Affiliations:** 1 Department of Periodontics, RAK College of Dental Sciences, RAK Medical & Health Sciences University, Ras-AlKhaimah 12973, United Arab Emirates; mustahsen@rakmhsu.ac.ae; 2 Department of Oral Rehabilitation, Faculty of Dentistry, University of Khartoum, Khartoum 11115, Sudan; 3 Department of Physiology, RAK College of Medical Sciences, RAK Medical & Health Sciences University, Ras-AlKhaimah 11172, United Arab Emirates; rashababiker@rakmhsu.ac.ae; 4 Department of Oral Surgery, RAK College of Dental Sciences, RAK Medical & Health Sciences University, Ras-AlKhaimah 12973, United Arab Emirates; riham.abdelraouf@rakmhsu.ac.ae; 5 Department of Pediatric and Preventive Dentistry, RAK College of Dental Sciences, RAK Medical & Health Sciences University, Ras-AlKhaimah 12973, United Arab Emirates; vivek.padmanabhan@rakmhsu.ac.ae; 6 Department of Operative Dentistry, RAK College of Dental Sciences, RAK Medical and Health Sciences University, Ras-AlKhaimah 12973, United Arab Emirates; sofiqul.islam@rakmhsu.ac.ae; 7 Department of Oral & Maxillofacial Surgery, Faculty of Dentistry, University of Khartoum, Khartoum 11115, Sudan; mariam.elsheikh@uofk.edu; 8 Department of Periodontology, Faculty of Dentistry, National University, Khartoum 11115, Sudan; salma.m.abduljalil@gmail.com; 9 Orthodontics Department, RAK College of Dental Sciences, RAK Medical & Health Sciences University, Ras-AlKhaimah 12973, United Arab Emirates; ghiathmahmoud@rakmhsu.ac.ae; 10 Department of Oral Medicine and Radiology, RAK College of Dental Sciences, RAK Medical & Health Sciences University, Ras-AlKhaimah 12973, United Arab Emirates; krishna.chytanya@rakmhsu.ac.ae; 11 Department of Oral Pathology, RAK College of Dental Sciences, RAK Medical & Health Sciences University, Ras-AlKhaimah 12973, United Arab Emirates; samarakon@rakmhsu.ac.ae; 12 Department of Periodontology and Implantology, Nile University, Khartoum 11115, Sudan; ayman.ahmed@nileuniversity.edu.sd

**Keywords:** periodontitis, antimicrobial resistance, biofilms, antimicrobial peptides, quorum sensing inhibitors, nanomedicine, host modulation, probiotics, postbiotics, pharmacogenomics

## Abstract

Periodontitis is a highly prevalent chronic inflammatory disease with significant oral and systemic consequences, including associations with cardiovascular disease, diabetes, and adverse pregnancy outcomes. Although mechanical debridement remains the cornerstone of therapy, adjunctive antibiotic use is increasingly limited by antimicrobial resistance, biofilm-associated tolerance, pharmacokinetic constraints, and disruption of the commensal microbiome, leading to inconsistent outcomes and disease recurrence. This review highlights the mechanistic limitations of conventional antibiotic therapies in periodontitis and critically examines emerging next-generation therapeutic strategies aimed at overcoming these challenges. Specifically, it explores antimicrobial peptides, quorum sensing inhibitors, nanotechnology-based drug delivery systems, host modulation approaches, and microbiome-targeted therapies, with emphasis on their molecular mechanisms, clinical relevance, and translational potential. By integrating microbial, host, and pharmacological perspectives, this review provides a comprehensive framework for advancing precision-guided periodontal therapy and supports the shift toward targeted, sustainable, and personalized treatment strategies.

## 1. Introduction

Periodontal diseases, particularly periodontitis, represent a global public health challenge with profound oral and systemic consequences. According to the Global Burden of Disease (GBD) Study, severe periodontitis is the sixth most prevalent disease worldwide, affecting nearly 800 million individuals and contributing substantially to disability-adjusted life years (DALYs) [1,2]. Periodontitis, in addition to merely being an oral disease, is a systemic health issue linked to major chronic conditions like cardiovascular disease, diabetes, respiratory diseases, and Alzheimer’s. It is a bidirectional relationship wherein these diseases can potentiate the development of one another. The successful treatment of periodontal disease can therefore improve overall health, demonstrating the crucial link between oral care and general well-being [2,3,4].

The consequences of periodontitis also extend to daily living and wellbeing through a direct reduction in oral health-related quality of life (OHRQoL) [5]. Patients with periodontitis frequently develop halitosis, gingival bleeding and eventually tooth mobility. These symptoms, along with masticatory dysfunction and potential tooth loss, severely compromise a patient’s ability to eat properly, communicate effectively, and maintain their self-esteem, thus impacting their nutrition, social life, and overall psychological health [6]. The S3-level clinical guidelines for periodontitis treatment from the European Federation of Periodontology further indicate that periodontitis alone is estimated to cost 54 billion dollars in direct treatment costs and 25 billion dollars in indirect costs [7].

The cumulative effect of these impairments highlighting the need for effective therapeutic strategies that address both microbial eradication and the host’s dysregulated inflammatory response [8]. Although mechanical plaque control through scaling and root planing remains the cornerstone of periodontal therapy, drug-based modalities are indispensable adjuncts in the suppression of bacterial load and the modification of host inflammatory pathways [9]. Antibiotics, such as tetracyclines, macrolides, nitroimidazoles, and penicillins, are frequently used in periodontology to treat periodontal disease by both killing bacteria and modulating the body’s immune response. They are administered systemically for widespread infections or locally for targeted action within deep periodontal pockets. The specific choice of antibiotic depends on the type of periodontal pathogen, dosage, and potential for bacterial resistance, with a focus on minimizing systemic side effects and overall antibiotic use for antimicrobial stewardship [10,11]. The effectiveness of antibiotics in periodontal treatments is limited by the increasing prevalence of antimicrobial resistance (AMR) among periodontal pathogens and the inherent ability of bacteria within biofilms to evade antibiotics [12]. Moreover, broad-spectrum antibiotic use disrupts both oral and gut microbiomes, potentially impairing long-term health and immune function by reducing microbial diversity [13]. This imbalance, or dysbiosis, can lead to pathogen overgrowth and is linked to chronic diseases like cardiovascular disease and inflammatory bowel disease [14,15]. Furthermore, treatment of periodontal infections with broad-spectrum antibiotics is hampered by inconsistent outcomes and high rates of disease recurrence [16]. These limitations have spurred research into new therapeutic strategies designed to complement or replace traditional antibiotics [17]. Recent years have witnessed the development of antimicrobial peptides, quorum sensing inhibitors, nanotechnology-based drug delivery systems, host modulation therapies, and microbiome engineering approaches, each offering promising avenues for overcoming the shortcomings of conventional regimens [18,19]. Nevertheless, progress in this field remains fragmented, with evidence dispersed across microbiology, pharmacology, nanomedicine, and clinical dentistry, limiting comprehensive translation into practice.

Although several recent reviews have discussed selected emerging therapies such as antimicrobial peptides, nanotechnology, probiotics, or host modulation, these topics are often addressed in isolation or from a broader anti-infective perspective rather than within the specific biological and clinical context of periodontitis [17,18,19]. A key unmet need in the literature is an integrated periodontal-focused analysis that links the mechanistic limitations of conventional antibiotics, particularly antimicrobial resistance, biofilm tolerance, pharmacokinetic constraints, microbiome disruption, and host-related variability—to the rationale, comparative advantages, and translational readiness of next-generation therapeutic strategies. In this context, the present review is intended not only to summarize emerging alternatives, but also to critically position them according to their biological targets, clinical relevance, and developmental maturity in periodontal therapy.

Therefore, this review aims to examine the mechanisms of conventional antibiotic therapies, to analyze the obstacles that restrict their effectiveness, to highlight advances in novel drug modalities with potential clinical applications, and to propose a future roadmap that integrates antimicrobial stewardship, precision medicine, and systemic health protection.

## 2. Mechanisms of Conventional Antibiotic in Periodontal Therapy

**Figure 1:**
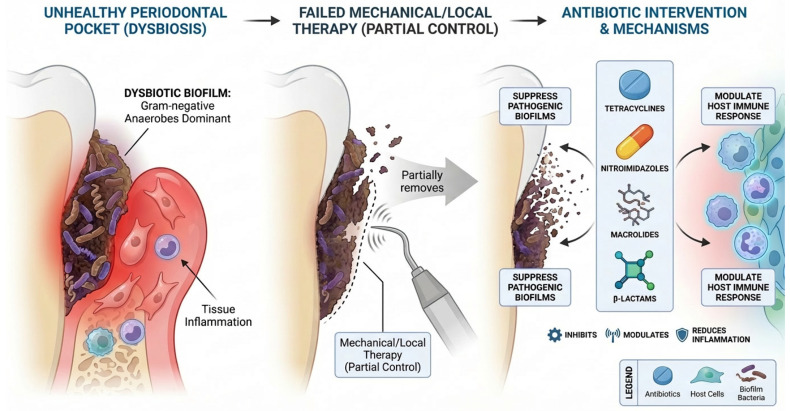
Mechanisms of conventional antibiotic therapies in periodontology. Tetracyclines inhibit protein synthesis via the 30S ribosomal subunit and suppress MMP-8/9, reducing collagen breakdown. Nitroimidazoles such as metronidazole generate nitro radicals in anaerobes, causing DNA strand breaks, with synergistic effects alongside amoxicillin. Macrolides bind the 50S ribosomal subunit, block peptide elongation, and reduce pro-inflammatory cytokines while concentrating in gingival crevicular fluid. β-lactams inhibit peptidoglycan cross-linking by binding PBPs, leading to bacterial lysis; however, all classes face limitations from resistance, biofilm resilience, and microbiome disruption. Figures were created using FigureLabs AI (https://www.figurelabs.ai), a web-based scientific illustration platform.

**Figure 2:**
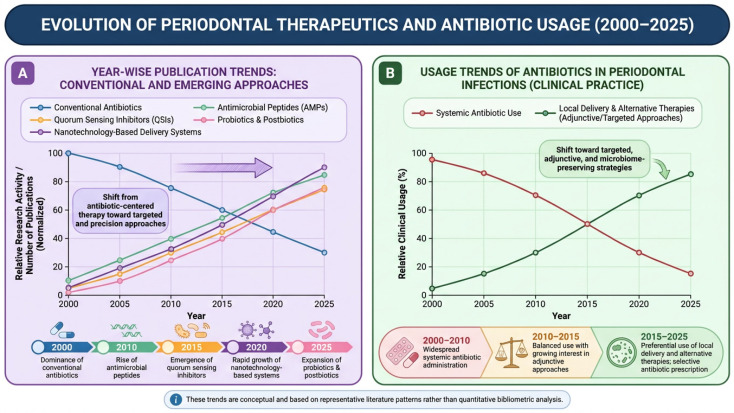
Evolution of periodontal therapeutics and antibiotic usage (2000–2025). (**A**) Schematic trends showing the shift from conventional antibiotics to emerging therapies, including AMPs, QSIs, nanotechnology-based systems, and microbiome-based approaches. (**B**) Conceptual trends in clinical antibiotic use demonstrating reduced reliance on systemic antibiotics and increased adoption of targeted and adjunctive therapies. Trends are schematic and based on representative literature patterns. Figure created with FigureLab.

Antibiotic use in periodontal therapy is justified by its microbial basis, in which a dysbiosis dominated by Gram-negative anaerobes emerges; when mechanical and local measures fail to control this shift, antibiotics are used to suppress the pathogenic load [20]. Conventional antibiotics like tetracyclines, nitroimidazoles, macrolides, and β-lactams suppress pathogenic biofilms and modulate host responses through multifaceted mechanisms beyond simply killing bacteria [21] (Figure 1 and Figure 2).

Tetracyclines function as bacteriostatic antibiotics by binding to the bacterial 30S ribosomal subunit, which prevents aminoacyl-tRNA from binding to the ribosomal A-site. This mechanism inhibits bacterial protein synthesis, halting bacterial growth and replication. The binding is reversible, and the antibiotic’s effect is most potent against actively multiplying bacteria [22]. This action suppresses the proliferation of periodontal pathogens such as Aggregatibacter actinomycetemcomitans (*A. actinomycetemcomitans*) and Porphyromonas gingivalis (*P. gingivalis*) [23,24]. A distinctive feature of tetracyclines, particularly doxycycline, is their ability to inhibit host matrix metalloproteinases (MMP-8 and MMP-9), enzymes responsible for collagen degradation and connective tissue breakdown. This dual antimicrobial and host-modulating effect forms the basis for the FDA-approved use of sub-antimicrobial dose doxycycline (20 mg, twice daily) as an adjunct in periodontal therapy [25,26].

Nitroimidazoles, most notably metronidazole, exert selective toxicity against anaerobic microorganisms. Upon entering the bacterial cell, the nitro group of metronidazole is reduced by electron transport proteins to form cytotoxic nitro radicals that bind to DNA, causing strand breakage and inhibition of nucleic acid synthesis [27]. This mechanism is particularly effective against obligate anaerobes such as *P. gingivalis* and Prevotella intermedia (*P. intermedia*), which are central to periodontal dysbiosis [28]. Adjunctive amoxicillin–metronidazole improves short-term results after scaling and root planing in stage III grade C periodontitis; metronidazole at 400–500 mg provides the greatest gains in clinical attachment level [29,30].

Macrolides, including azithromycin and erythromycin, function by binding to the 50S ribosomal subunit to inhibit protein synthesis and block the elongation of the growing peptide chain [31]. Their activity extends beyond antibacterial effects, as azithromycin exhibits potent immunomodulatory properties, reducing the expression of pro-inflammatory cytokines such as IL-1β, TNF-α, and IL-6, while also interfering with biofilm maturation [32]. Importantly, azithromycin demonstrates high gingival crevicular fluid concentrations, enabling sustained antimicrobial activity within the periodontal pocket [33].

β-lactam antibiotics, such as amoxicillin, function by inhibiting bacterial cell wall synthesis. They do this by irreversibly binding to and inactivating penicillin-binding proteins (PBPs), which are enzymes essential for the cross-linking of peptidoglycan strands that provide structural integrity to the bacterial cell wall. This disruption leads to a weakened cell wall, causing the bacterial cell to lyse and die [34]. Amoxicillin is often used in combination with metronidazole, yielding a synergistic effect against *A. actinomycetemcomitans P. gingivalis*. This combined antibiotic therapy can achieve better clinical and microbiological outcomes than monotherapy alone, helping to improve periodontal conditions and reduce harmful bacteria [35,36]. Collectively, these conventional antibiotics remain clinically relevant as adjuncts to mechanical debridement, offering microbial suppression, anti-inflammatory benefits, and improved clinical attachment levels. However, their success is increasingly compromised by several obstacles.

## 3. Obstacles Restricting the Effectiveness of Conventional Antibiotics

Despite their established clinical roles, conventional antibiotic therapies in periodontology face fundamental limitations at the molecular and ecological levels that compromise long-term efficacy [37]. A critical obstacle is the emergence of antimicrobial resistance (AMR) among periodontal pathogens. A retrospective study of 7804 periodontitis patients revealed that 63.5% of cases developed resistance to at least one antibiotic. Notably, antibiotic resistance rates surged from 37% in 2008 to 70% in 2015 [38].

**Figure 3:**
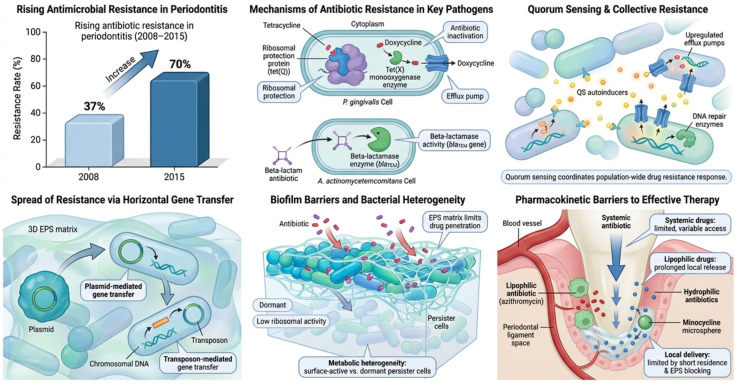
This schematic summarizes the key factors driving antimicrobial resistance in periodontal infections. The rising prevalence of resistance reflects widespread antibiotic use. Periodontal pathogens employ multiple mechanisms, including enzymatic drug degradation, target modification, reduced membrane permeability, and active efflux, which collectively decrease intracellular antibiotic concentrations. At the community level, quorum sensing and coordinated bacterial behavior promote biofilm formation and adaptive resistance. Horizontal gene transfer—via plasmids, transposons, and bacteriophages—facilitates rapid dissemination of resistance genes, while biofilm-associated barriers limit drug penetration and support persister cells with reduced susceptibility. Additionally, pharmacokinetic constraints within periodontal pockets may restrict effective drug delivery. Together, these factors reduce antibiotic efficacy and highlight the need for targeted and alternative therapeutic strategies. Figure created with FigureLab.

Antibiotic resistance spreads rapidly in the oral microbiome via horizontal gene transfer (HGT), primarily through plasmids and transposons that move resistance determinants between bacteria. Plasmids are mobile genetic elements that facilitate the exchange of genes for resistance, while transposons are “jumping genes” that can move resistance genes between different DNA molecules [39]. This HGT process is particularly efficient in oral biofilms, where bacteria are in close proximity, allowing for the quick dissemination of antibiotic resistance among diverse bacterial species in the mouth [39]. For example, *P. gingivalis* resists tetracyclines through mechanisms that prevent the drug from reaching its target on the ribosome or by directly inactivating it. Specifically, the tet(Q) gene produces ribosomal protection proteins that dislodge tetracyclines from the 30S ribosome, while the tet(X) gene encodes monooxygenases that enzymatically inactivate doxycycline. Additionally, efflux pumps, such as Resistance–Nodulation–Division (RND) family transporters, actively remove these antibiotics from the bacterial cell, reducing their intracellular concentration [40,41] (Figure 3).

In *A. actinomycetemcomitans*, β-lactam resistance arises from the acquisition of blaTEM β-lactamase genes, which hydrolyze the β-lactam ring of penicillin derivatives, thereby neutralizing amoxicillin [42,43]. These molecular mechanisms implicate that even high systemic doses of antibiotics may be ineffective against resistant subpopulations, contributing to treatment failure and recurrence [44]. Another major barrier is the biofilm phenotype of periodontal infections, which are communities of microbes embedded in a protective matrix of extracellular polymeric substances (EPS) [45]. This EPS matrix acts as a physical barrier, reducing the penetration of antibiotics like metronidazole, and contains components that can increase the bacteria’s resistance to medications and the body’s immune system [46]. Additionally Within the biofilm, bacteria display metabolic heterogeneity: actively dividing cells near the surface remain susceptible to antibiotics, while oxygen-deprived, nutrient-limited persister cells in the deeper layers shift into dormancy. These persisters exhibit reduced ribosomal activity and altered proton motive force, rendering ribosome-targeting antibiotics such as tetracyclines and macrolides ineffective [47] (Figure 3).

Quorum sensing (QS) allows bacteria to communicate their population density, leading to collective responses that enhance their resistance to antibiotics. When bacteria are stressed, such as by the presence of antibiotics, QS triggers the collective upregulation of efflux pumps, which expel antibiotics from the cell, and DNA repair enzymes, which fix any damage to the bacterial DNA caused by antibiotics. These coordinated actions collectively blunt the effect of the antibiotic, helping the bacterial population to survive and thrive in the presence of the drug [48,49]. Pharmacokinetic limitations also restrict antibiotic access to periodontal niches. Systemic administration may not consistently achieve therapeutic levels in gingival crevicular fluid due to protein binding, rapid clearance, and variable tissue diffusion [50]. Lipophilic antibiotics like azithromycin can concentrate within gingival cells and phagocytes, enabling sustained release into periodontal pockets, whereas hydrophilic drugs struggle to accumulate. Local delivery systems such as minocycline microspheres and doxycycline gels improve drug delivery to the site but face limitations including short residence times, enzymatic breakdown, and difficulty reaching deep into complex periodontal pockets [51,52] (Figure 3).

**Figure 4:**
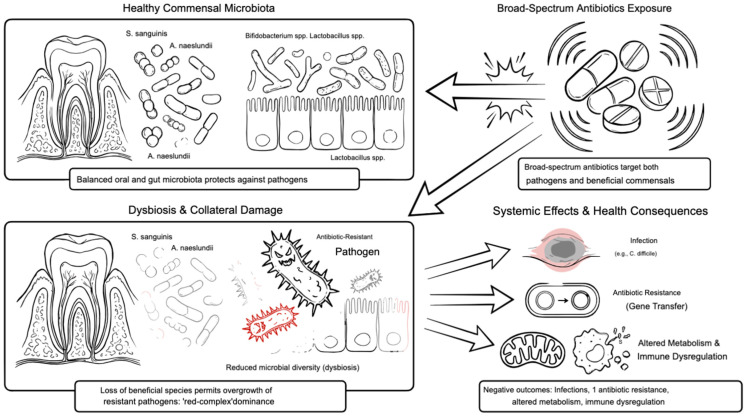
Collateral damage of broad-spectrum antibiotics on commensal microbiota. Beneficial oral species such as *Streptococcus sanguinis* and Actinomyces naeslundii are eliminated, destabilizing microbial homeostasis and permitting recolonization by red-complex pathogens, leading to dysbiosis. Systemic antibiotic exposure also disrupts the gut microbiome, reducing diversity and short-chain fatty acid production, weakening epithelial barrier function, and amplifying systemic inflammation. This collateral damage favors recurrent periodontitis and broader immune dysregulation. Figure created with FigureLab.

The use of broad-spectrum antibiotics causes a disruption to the beneficial commensal microbiota, leading to dysbiosis. This “collateral damage” involves reduced microbial diversity and the overgrowth of opportunistic, antibiotic-resistant pathogens that were previously kept in check by the healthy microbiota. This disruption can lead to a range of negative health consequences, including an increased risk of infection, the development of antibiotic resistance, and altered metabolic processes [53]. Disruption of beneficial species such as *Streptococcus sanguinis* (*S. sanguinis*) and Actinomyces naeslundii (*A. naeslundii*) destabilizes microbial homeostasis, eliminating ecological competitors that normally suppress the overgrowth of pathogens. This ecological void permits the re-establishment of virulent consortia dominated by red-complex organisms [54,55]. Dysbiosis is not confined to the oral cavity; systemic antibiotics also perturb the gut microbiome, with downstream effects on metabolism and immune regulation [56] (Figure 4).

**Figure 5:**
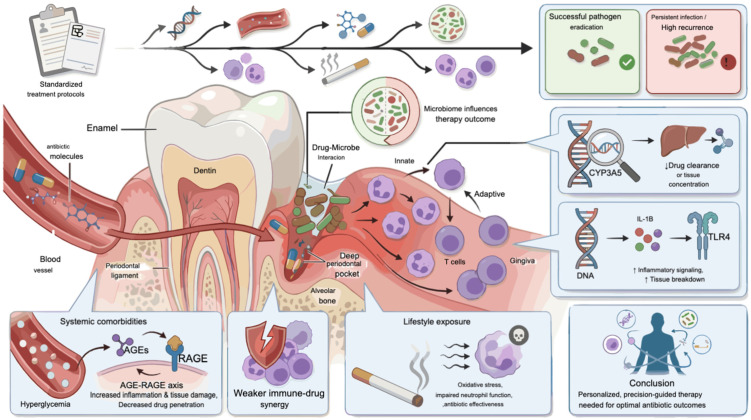
Host-related variability in antibiotic outcomes. Genetic polymorphisms affecting drug metabolism (e.g., CYP3A5) and inflammatory responses (e.g., IL-1β, TLR4) alter therapeutic responsiveness. Systemic conditions such as diabetes and immunocompromised states further reduce drug delivery and immune synergy, while lifestyle factors like smoking exacerbate oxidative stress and impair healing. Collectively, these host-dependent factors lead to reduced antibiotic efficacy and high recurrence of periodontitis. Figure created with FigureLab.

Furthermore, clinical variability in how well antibiotics work shows the significant role the host’s physiology and immune system plays in determining drug effectiveness. The effectiveness of an antibiotic isn’t just about the drug and the microbe; the host’s ability to fight the infection, the health of both innate and adaptive immune system, and the balance of host microbiome all interact with the antibiotic therapy to influence outcomes [57,58]. Genetic polymorphisms significantly influence both pharmacokinetics and inflammatory responsiveness. For instance, variants in CYP3A5 alter azithromycin clearance, determining whether therapeutic concentrations are achieved in gingival crevicular fluid, while polymorphisms in IL-1β and TLR4 enhance inflammatory signaling, predisposing certain individuals to persistent tissue breakdown despite bacterial suppression [59,60]. Systemic comorbidities further shape therapeutic response. Diabetes mellitus negatively affects therapeutic outcomes by both reducing the penetration of medications into periodontal tissues due to impaired microvascular perfusion and by amplifying the pro-inflammatory AGE-RAGE signaling pathway, which perpetuates chronic inflammation and tissue damage in periodontal disease. This dual impact creates a vicious cycle where hyperglycemia leads to increased AGEs, which in turn activate RAGE, causing further inflammation, oxidative stress, and tissue destruction, making periodontal treatments less effective [61,62]. Similarly, immunocompromised states or cardiovascular disease may blunt immune-drug synergy, weakening therapeutic gains. Lifestyle factors, particularly smoking, exacerbate oxidative stress and dysregulate neutrophil function, diminishing antibiotic efficacy and delaying wound healing [63,64]. Even under standardized clinical protocols, these layers of host-specific variability limit pathogen eradication and contribute to the high recurrence rates observed in periodontitis, highlighting the necessity for precision-guided, personalized therapeutic approaches [65] (Figure 5).

Together, these molecular, pharmacological, and ecological barriers highlight why antibiotics alone cannot constitute a curative solution for periodontal infections. They reinforce the need for alternative drug modalities that circumvent resistance mechanisms, penetrate biofilms, preserve microbial ecology, and adapt to host variability.

## 4. Emerging Therapeutic Strategies Beyond Conventional Antibiotics

The limitations of traditional antibiotics in periodontal therapy such as increasing bacterial resistance and biofilm protection, have spurred research into alternative periodontal therapies that target bacterial virulence, biofilm resilience, and host inflammatory pathways. Several promising avenues have emerged, ranging from naturally derived antimicrobial peptides to sophisticated nanotechnology-based delivery systems. Each strategy exploits unique molecular mechanisms that may circumvent resistance and improve therapeutic precision.

### 4.1. Antimicrobial Peptides (AMPs)

Antimicrobial peptides (AMPs) are considered promising alternatives to conventional antibiotics in periodontal therapy because they are naturally produced by organisms and offer a first line of defense against pathogens [66]. They are less likely to induce resistance, can modulate the host’s immune response, and offer a broad spectrum of activity, making them valuable in combating drug-resistant bacteria and treating periodontal disease (Table 1) [67]. However, challenges such as susceptibility to degradation by saliva enzymes and potential host cell toxicity need to be addressed for their widespread clinical adoption [68]. AMPs are short, cationic, amphipathic molecules that exert broad-spectrum activity against Gram-negative and Gram-positive bacteria, fungi, and even some viruses [68]. Their mechanism of action is primarily membrane-targeting rather than enzyme- or protein-specific, making resistance development significantly less likely [68]. In the context of periodontology, AMPs not only neutralize periodontal pathogens such as *P. gingivalis*, *A. actinomycetemcomitans*, and Tannerella forsythia (*T. forsythia*) but also modulate the host immune response in ways that favor tissue homeostasis and wound healing [69,70].

#### 4.1.1. Molecular Mechanisms of AMPs

**Figure 6:**
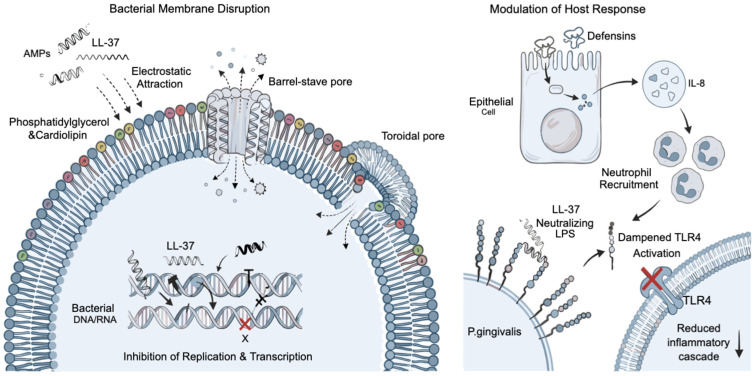
Antimicrobial and immunomodulatory mechanisms of LL-37 in periodontal and peri-implant environments. LL-37 disrupts bacterial membranes through electrostatic interactions with negatively charged phospholipids, leading to pore formation (barrel-stave and toroidal mechanisms) and membrane destabilization. In addition, LL-37 penetrates bacterial cells and inhibits DNA/RNA replication and transcription. On the host side, LL-37 modulates immune responses by influencing epithelial cell signaling, promoting IL-8–mediated neutrophil recruitment, and neutralizing bacterial lipopolysaccharide (LPS), thereby reducing Toll-like receptor 4 (TLR4) activation and dampening inflammatory cascades. Arrows indicate the direction of biological processes, while inhibitory effects are indicated by red cross symbols. The DNA/RNA structures are shown schematically and do not represent specific nucleotide sequences. Figure created with FigureLab.

AMPs disrupt bacterial membranes through electrostatic interactions with negatively charged phospholipids such as phosphatidylglycerol and cardiolipin, which are enriched in bacterial membranes but rare in mammalian cells [82]. After initial attraction, AMPs insert into the lipid bilayer and form transmembrane pores (barrel-stave or toroidal pore models), causing leakage of cytoplasmic contents and rapid bacterial death. Some AMPs, such as LL-37, additionally intercalate with bacterial DNA and RNA, inhibiting replication and transcription [72,83]. Importantly, AMPs can modulate the host response: defensins upregulate IL-8 expression in epithelial cells to enhance neutrophil recruitment, while LL-37 binds to lipopolysaccharides (LPS) of *P. gingivalis*, neutralizing endotoxin-mediated Toll-like receptor 4 (TLR4) activation and dampening the excessive inflammatory cascade [73,84] (Figure 6).

#### 4.1.2. Endogenous AMPs in Periodontal Health and Disease

In the healthy periodontium, human β-defensins (hBD-1, hBD-2, hBD-3) and cathelicidin LL-37 are constitutively expressed by gingival epithelial cells, salivary glands, and neutrophils [85]. hBD-2 and hBD-3 are upregulated during periodontal infection, serving as a first-line defense against invading pathogens. However, in chronic periodontitis, persistent bacterial virulence factors can suppress AMP gene expression via NF-κB modulation, undermining innate immunity [86]. This paradoxical reduction of AMP levels in diseased gingiva highlights their potential therapeutic supplementation.

#### 4.1.3. Synthetic and Modified AMPs

Several synthetic and semi-synthetic AMPs have been designed to improve stability and reduce cytotoxicity while retaining antimicrobial potency [87].

- Pexiganan, a synthetic analog of magainin, exhibits strong bactericidal activity against *P. gingivalis* and *Prevotella intermedia* and has been tested as a topical gel in periodontal pockets [88].
- Novispirin, derived from ovispirin, demonstrates reduced hemolytic activity and enhanced selectivity toward Gram-negative pathogens [89].
- Omiganan (MBI 226) has shown biofilm-disruptive activity and anti-inflammatory properties, making it a candidate for adjunctive periodontal therapy [90].
- D-enantiomeric AMPs (composed of D-amino acids rather than L-amino acids) resist proteolytic degradation in the periodontal pocket, prolonging their half-life in inflamed environments rich in host and bacterial proteases [91,92].

#### 4.1.4. Nanostructured AMP Delivery

Given the short half-life and rapid degradation of peptides in vivo, research has focused on nanoparticle-based delivery systems. AMPs encapsulated in chitosan or PLGA nanoparticles demonstrate enhanced stability, controlled release, and improved penetration into periodontal biofilms [93]. For instance, LL-37-loaded nanocarriers achieved sustained antibacterial activity and enhanced gingival fibroblast migration, supporting tissue healing. Self-assembling AMP hydrogels are also under investigation as injectable, bioadhesive formulations for deep periodontal pockets [94,95].

#### 4.1.5. Dual Antimicrobial and Host-Modulating Action

Unlike antibiotics, AMPs act at the interface of antimicrobial and host-modulatory pathways [73]. LL-37 not only kills *A. actinomycetemcomitans* but also stimulates angiogenesis via vascular endothelial growth factor (VEGF) induction, promoting periodontal regeneration [73,96]. β-defensins influence osteoclast differentiation through the RANKL/OPG axis, potentially modulating alveolar bone loss [97]. These dual functions make AMPs particularly attractive in a disease like periodontitis, where tissue destruction is driven as much by the host inflammatory response as by microbial virulence.

Overall, AMPs are particularly promising because they combine broad antimicrobial activity with host-modulatory functions, a dual property highly relevant to periodontitis. However, despite these advantages, their clinical translation remains less advanced than their mechanistic rationale would suggest, mainly because instability in the oral environment, formulation challenges, cost, and potential cytotoxicity continue to limit routine application.

### 4.2. Quorum Sensing Inhibitors (QSIs)

Periodontal pathogens rely heavily on quorum sensing (QS), a cell–cell communication system mediated by diffusible signaling molecules, to coordinate virulence gene expression, biofilm maturation, and immune evasion [98]. Quorum sensing inhibitors (QSIs) are an emerging class of antimicrobials that, unlike conventional antibiotics, disrupt bacterial communication by blocking their QS systems [99]. This strategy allows pathogens to maintain their virulence and other functions without killing the bacteria, thereby avoiding the strong selective pressure that promotes the development of antibiotic resistance [100]. Instead, by attenuating virulence factors and biofilm formation, QSIs make bacteria more susceptible to the host’s immune system, or they can be used in conjunction with conventional antibiotics to improve their efficacy [99,100].

#### 4.2.1. Molecular Basis of Quorum Sensing in Periodontal Pathogens

**Figure 7:**
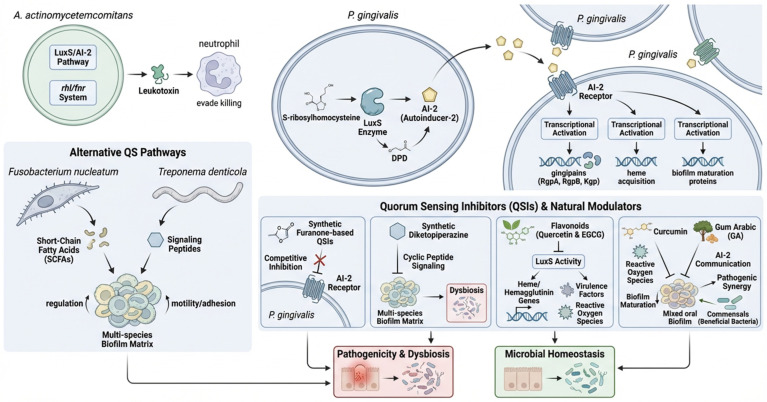
LuxS/AI-2-mediated quorum sensing in Porphyromonas gingivalis and Aggregatibacter actinomycetemcomitans and its role in periodontal dysbiosis and microbial homeostasis. In both species, the LuxS enzyme catalyzes the conversion of S-ribosylhomocysteine into the AI-2 signaling molecule via the intermediate DPD. AI-2 is released into the extracellular environment, where it accumulates and is sensed by bacterial cells, leading to activation of quorum sensing–dependent transcriptional programs. In P. gingivalis, AI-2 signaling regulates the expression of key virulence factors, including gingipains (RgpA, RgpB, Kgp), heme acquisition systems, and biofilm-associated proteins, thereby promoting biofilm maturation and pathogenicity. In A. actinomycetemcomitans, quorum sensing pathways, including the LuxS/AI-2 system and the rhl/fnr regulatory system, contribute to leukotoxin production and enhanced evasion of neutrophil-mediated killing. Alternative quorum sensing pathways in other periodontal pathogens, such as Fusobacterium nucleatum and Treponema denticola, involve short-chain fatty acids and signaling peptides that further support multispecies biofilm formation, adhesion, and motility. Quorum sensing inhibitors (QSIs) and natural modulators, including furanone-based compounds, diketopiperazines, flavonoids (e.g., quercetin and EGCG), curcumin, and plant-derived compounds, interfere with AI-2 signaling through receptor inhibition, competitive binding, or modulation of signaling pathways. These effects result in reduced virulence factor expression, disruption of biofilm formation, and restoration of microbial balance. Collectively, activation of quorum sensing pathways contributes to periodontal dysbiosis and increased pathogenicity, whereas inhibition of these pathways promotes microbial homeostasis. Arrows indicate the direction of signaling pathways and biological processes. Figure created with FigureLab.

In *P. gingivalis*, quorum sensing is primarily mediated through the LuxS/autoinducer-2 (AI-2) pathway, a conserved interspecies communication system. This system’s signal, AI-2, is produced from S-ribosylhomocysteine by the LuxS enzyme, which cleaves it into 4,5-dihydroxy-2,3-pentanedione (DPD), a precursor that then cyclizes to form AI-2 [101]. This process is part of the activated methyl cycle and plays a crucial role in the bacterium’s survival and pathogenicity in the oral environment [101] (Figure 7). Upon secretion, AI-2 is sensed by neighboring bacterial cells, leading to transcriptional activation of genes involved in gingipain production (RgpA, RgpB, Kgp), heme acquisition systems, and biofilm maturation proteins. In *A. actinomycetemcomitans*, QS is coordinated by the LuxS/AI-2 system and the rhl/fnr system, which regulate leukotoxin expression, enhancing the pathogen’s capacity to evade neutrophil-mediated killing [102] (Figure 7).

#### 4.2.2. Mechanistic Action of Quorum Sensing Inhibitors

Synthetic furanone-based QSIs that mimic Autoinducer-2 (AI-2) signal molecules act by competitively blocking AI-2 receptors, preventing bacterial communication, and inhibiting the expression of virulence genes, such as gingipains in *P. gingivalis* [103]. This disrupts the formation and development of bacterial biofilms and reduces the secretion of harmful enzymes and toxins, thereby mitigating pathogenicity [103]. Flavonoids, such as quercetin and epigallocatechin gallate (EGCG), and the polyphenol curcumin have been shown to inhibit bacterial QS and reduce oxidative stress caused by pathogens. Quercetin and EGCG work by interfering with LuxS activity, which is involved in AI-2 signaling, and by downregulating genes that help bacteria acquire heme and produce hemagglutinin. Curcumin also targets AI-2 signaling and reduces oxidative stress pathways, providing a dual protective effect against periodontal pathogens [104]. Beyond the common LuxS/AI-2 QS system, other signaling circuits are recognized, including short-chain fatty acids (SCFAs) from Fusobacterium nucleatum and signaling peptides from Treponema denticola, which regulate multi-species biofilms and motility/adhesion. QSIs like synthetic diketopiperazines can target these alternative pathways, acting as competitive inhibitors for cyclic peptide signaling and disrupting community interactions that contribute to dysbiosis [105]. In addition to synthetic QSIs, natural plant-derived compounds such as gum Arabic (GA) have shown promising modulatory effects on quorum sensing. GA, a biopolymer rich in arabinogalactan-proteins, not only exhibits prebiotic properties but also interferes with bacterial signaling cascades [106] (Figure 7). Recent findings suggest that GA can attenuate AI-2 mediated communication, thereby reducing biofilm maturation and pathogenic synergy among oral bacteria. Its dual role supporting beneficial commensals while dampening pathogenic quorum sensing, positions GA as a potential adjunctive strategy for maintaining microbial homeostasis and counteracting periodontal dysbiosis [106].

#### 4.2.3. Intracellular Pathway Interference

At the intracellular level, QSI-mediated blockade of AI-2 prevents activation of the two-component signal transduction systems (e.g., LsrR/LsrK in *A. actinomycetemcomitans*), which normally phosphorylate AI-2 and induce QS-regulated transcription [107]. Inhibition of these systems downregulates expression of genes encoding toxins, proteases, and adhesion molecules [107].

QSIs can indeed interfere with the Rhl/FNR pathway, which connects bacterial communication to oxygen levels and metabolic processes, ultimately hindering the survival of pathogens like *P. gingivalis* in the low-oxygen (hypoxic) environments found in periodontal pockets [108]. By disrupting this signaling system, QSIs can reduce bacterial virulence and biofilm formation, offering a potential therapeutic strategy for managing periodontitis without causing antibiotic resistance [108,109].

#### 4.2.4. Therapeutic Implications in Periodontology

QSIs hold unique advantages in periodontal therapy. By dismantling bacterial communication rather than killing cells, they reduce the selective pressure for resistance while synergizing with host defenses and conventional antimicrobials [110]. For instance, Furanones are a class of QSIs that can disrupt bacterial biofilm formation, making biofilms more vulnerable to antibiotics like doxycycline by enhancing their permeability and enabling effective dual action at lower antibiotic doses [111]. QSIs like furanones may also help maintain oral microbial homeostasis by selectively disrupting pathogenic bacterial communication while preserving beneficial commensal bacteria, which often possess different signaling systems [111].

To provide a more translational and up-to-date overview, Table 2 summarizes representative synthetic and natural quorum sensing inhibitors relevant to periodontology, highlighting their molecular targets, reported inhibitory concentrations where available, periodontal relevance, and current level of evidence. Recent reviews indicate that this field has expanded beyond a few prototype molecules, making a comparative and data-enriched summary necessary.

From a conceptual standpoint, QSIs represent one of the most resistance-conscious approaches because they attenuate virulence without directly killing bacteria. However, their current evidence base remains predominantly preclinical, and uncertainty persists regarding durability of effect in complex multispecies oral biofilms, optimal delivery systems, and the extent to which quorum sensing blockade alone can produce clinically meaningful periodontal improvement.

### 4.3. Nanotechnology-Based Drug Delivery

**Figure 8:**
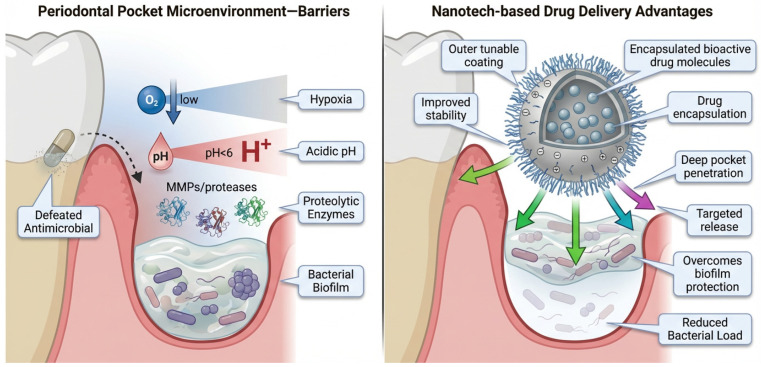
Periodontal pocket microenvironment barriers and advantages of nanotechnology-based drug delivery systems. The left panel illustrates the complex microenvironment of the periodontal pocket, characterized by hypoxia (low oxygen levels), acidic pH (pH < 6), and elevated levels of matrix metalloproteinases (MMPs) and proteolytic enzymes. These conditions promote the formation of a dense bacterial biofilm and contribute to degradation and inactivation of conventional antimicrobial agents, thereby reducing therapeutic efficacy. The right panel demonstrates how nanotechnology-based drug delivery systems can overcome these limitations. Encapsulation of bioactive agents enhances drug stability and protects against enzymatic degradation, while tunable surface coatings and targeting capabilities improve localization within periodontal tissues. Nanoparticles facilitate deeper penetration into the periodontal pocket and biofilm matrix, enabling more effective drug delivery. Additionally, these systems can bypass biofilm-associated resistance mechanisms, leading to reduced bacterial load and improved therapeutic outcomes. Arrows indicate the direction of drug delivery and biological interactions within the periodontal pocket. Figure created with FigureLab.

The unique microenvironment of periodontal pockets characterized by hypoxia, acidic pH, proteolytic enzymes, and a biofilm-protected bacterial consortium poses significant challenges to conventional antimicrobials [125]. Nanotechnology-based drug delivery systems are designed to overcome these barriers by improving drug stability, penetration, and targeted release. Their small size, surface tunability, and ability to encapsulate bioactive molecules provide distinct advantages for controlling bacterial infections in periodontology [126] (Figure 8).

#### 4.3.1. Polymeric Nanoparticles (NPs)

Biodegradable polymers such as poly(lactic-co-glycolic acid) (PLGA) and chitosan are widely employed for antibiotic delivery. PLGA nanoparticles encapsulating doxycycline or metronidazole enable sustained release within the periodontal pocket for several weeks, achieving therapeutic concentrations without systemic toxicity [127]. Chitosan, a cationic polysaccharide, exhibits inherent antimicrobial activity by interacting with negatively charged bacterial membranes, causing leakage of intracellular components. When used as a carrier for tetracyclines, chitosan nanoparticles enhance drug penetration into biofilms and prolong antibacterial effects [128].

#### 4.3.2. Metallic Nanoparticles

Silver nanoparticles (AgNPs) and zinc oxide nanoparticles (ZnO-NPs) in periodontology exert antimicrobial effects through ROS generation, oxidative damage to cellular components, and the release of silver and zinc ions, which disrupt membrane integrity and inhibit cellular processes [129]. These nanoparticles also show promise in targeting pathogenic biofilms, a significant challenge in periodontology, by inhibiting their formation and potentially breaking down existing structures. Their combined effects offer a potent strategy for combating periodontitis-causing bacteria by addressing multiple attack points within the microorganisms [130]. Silver nanoparticles have demonstrated strong inhibitory activity against *P. gingivalis* and *A. actinomycetemcomitans*, including biofilm-associated cells that typically evade antibiotics. Importantly, metallic NPs can be incorporated into local delivery gels or biodegradable films for site-specific application [131].

#### 4.3.3. Lipid-Based Nanocarriers

Liposomes and Solid Lipid Nanoparticles (SLNs) are advanced drug delivery systems used in periodontal therapy, mimicking biological membranes to effectively encapsulate and deliver both hydrophilic and hydrophobic drugs [132]. Liposomes excel at holding hydrophilic drugs in their inner aqueous core and hydrophobic drugs in their lipid bilayer, while SLNs offer a lipid matrix for hydrophobic drug containment [133]. This dual capacity for drug loading, coupled with their biocompatibility, controlled release properties (demonstrated by doxycycline in liposomes and chlorhexidine in SLNs), and ability to interact with bacterial membranes, makes them highly promising for treating periodontal diseases [134].

#### 4.3.4. Stimuli-Responsive and Targeted Systems

Recent advancements in periodontal therapy focus on smart nanoparticles that respond to the specific chemical and physical cues within periodontal pockets to deliver drugs, control infection, and promote tissue regeneration [135]. These nanomaterials can release therapeutic agents in a targeted manner, adapting to the unique pathological microenvironment of periodontitis by responding to stimuli such as pH, inflammation markers, or temperature. This controlled release improves the efficiency of treatment and has the potential to overcome the limitations of traditional periodontal therapies by promoting site-specific and dose-specific drug delivery, ultimately enhancing therapeutic outcomes and patient compliance [136]. For instance, pH-sensitive nanoparticles release drugs selectively in acidic conditions characteristic of periodontal inflammation, maximizing therapeutic efficiency while sparing healthy sites. Enzyme-responsive nanocarriers, designed to degrade in the presence of gingipains or host-derived proteases, enable site-activated release of antimicrobials. Surface functionalization with targeting ligands, such as antibodies against *P. gingivalis* fimbriae, further enhances specificity and reduces off-target effects [137].

#### 4.3.5. Nanoparticle-Delivered Novel Agents

**Figure 9:**
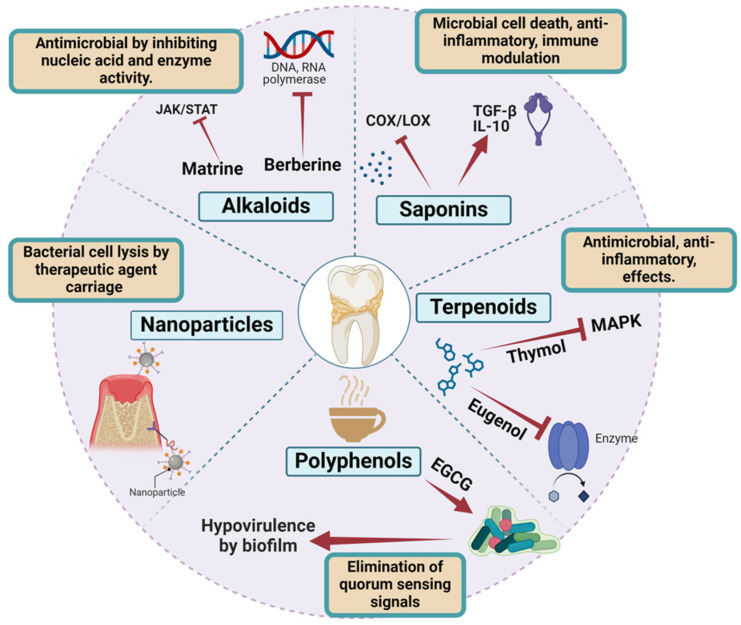
Mechanisms of action of natural compounds and nanotechnology-based agents in periodontal therapy. The figure illustrates the major classes of bioactive compounds, including alkaloids, saponins, terpenoids, and polyphenols, along with nanotechnology-based delivery systems, and their roles in modulating periodontal disease processes. Alkaloids exhibit antimicrobial activity by inhibiting nucleic acid synthesis and enzyme function, while saponins contribute to microbial cell death and modulation of inflammatory and immune responses. Terpenoids demonstrate antimicrobial and anti-inflammatory effects through regulation of signaling pathways such as MAPK. Polyphenols exert multiple actions, including biofilm disruption, elimination of quorum sensing signals, and reduction of oxidative stress. Nanoparticles enhance therapeutic efficacy by improving drug stability, facilitating targeted delivery, and increasing penetration into biofilms and periodontal tissues. Arrows indicate the direction of biological processes and signaling pathways, while dashed arrows represent indirect or modulatory effects [139].

Nanocarriers enhance antimicrobial therapies by encapsulating antimicrobial peptides (AMPs) and natural compounds, such as curcumin, into stable polymeric nanoparticles for increased efficacy. AMPs protected by nanocarriers resist degradation, extending their therapeutic lifespan. Nano-formulated polyphenols like curcumin demonstrate improved solubility and stability, leading to significant biofilm reduction in experimental models. This nanotechnology approach improves drug delivery, protects active molecules from breakdown, and enhances overall therapeutic potential against infections and antibiotic-resistant bacteria [138,139] (Figure 9). In regenerative approaches, nanoparticles delivering statins or growth factors promote osteoblastic differentiation and periodontal regeneration while simultaneously suppressing infection [140].

#### 4.3.6. Clinical and Translational Perspectives

While preclinical studies highlight strong antimicrobial and host-modulatory potential, clinical translation remains limited. Concerns include nanoparticle cytotoxicity at higher doses, variability in drug release kinetics, and regulatory challenges regarding biocompatibility. Nevertheless, ongoing clinical trials are investigating doxycycline- and chlorhexidine-loaded nanocarriers for local periodontal therapy, and early results suggest improved pocket depth reduction and attachment gain compared to conventional formulations [141,142].

Compared with other emerging modalities, nanotechnology may be among the most immediately translatable because it can enhance the performance of existing therapeutic agents rather than requiring entirely new pharmacological classes. Its main advantage lies in solving pharmacokinetic and biofilm-penetration barriers; however, its real-world adoption will depend on demonstrating reproducible safety, scalable manufacturing, and clinically meaningful superiority over currently available local delivery systems.

### 4.4. Host Modulation Therapy

**Figure 10:**
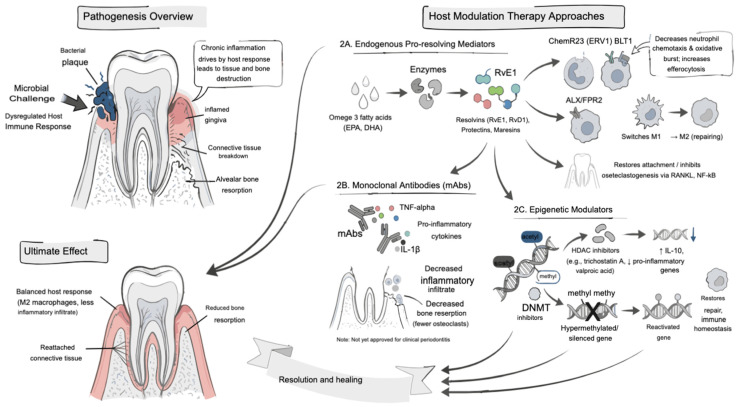
Host modulation therapy strategies in the management of periodontal inflammation. This schematic illustrates the role of host modulation therapy (HMT) in controlling periodontal inflammation and promoting tissue healing. Periodontitis is characterized by a dysregulated host immune response to bacterial biofilms, leading to connective tissue breakdown and alveolar bone resorption. Three major HMT approaches are highlighted. (**2A**) Endogenous pro-resolving mediators, including lipoxins, resolvins, and maresins, actively regulate the resolution phase of inflammation by reducing neutrophil infiltration, enhancing macrophage-mediated clearance, and promoting tissue repair. (**2B**) Monoclonal antibodies (mAbs) target key pro-inflammatory cytokines such as TNF-α and IL-1β, thereby reducing inflammatory signaling, inhibiting osteoclast activity, and limiting bone loss. (**2C**) Epigenetic modulators influence gene expression through mechanisms such as histone modification and DNA methylation, suppressing pro-inflammatory pathways while promoting regenerative processes. Collectively, these approaches shift the host response from chronic inflammation toward resolution and healing, resulting in improved periodontal stability and reduced tissue destruction. Arrows indicate the direction of biological processes, signaling pathways, and therapeutic effects. Figure created with FigureLab.

The progression of periodontitis is driven not only by microbial challenge but also by an exaggerated and dysregulated host immune response [143]. Host Modulation Therapy (HMT) manages periodontitis by targeting the host’s immune response to reduce tissue damage, unlike direct antimicrobials which target bacteria. In diseases like periodontitis, the unchecked inflammatory response causes connective tissue breakdown and alveolar bone resorption [144]. HMT aims to prevent tissue destruction, restore a healthy balance between the immune system and microbes, and promote the resolution of inflammation, ultimately leading to periodontal healing. This therapy works by modulating inflammatory pathways, influencing immune cells and inflammatory mediators to reduce destruction while preserving the ability to fight infection [145,146] (Figure 10).

#### 4.4.1. Specialized Pro-Resolving Lipid Mediators (SPMs)

Resolvins (RvE1, RvD1), protectins, and maresins are biosynthesized from omega-3 fatty acids (EPA, DHA) during the resolution phase of inflammation [147]. RvE1 binds to ChemR23 (ERV1 receptor) and BLT1 (leukotriene B4 receptor) on neutrophils and macrophages, suppressing neutrophil chemotaxis and oxidative burst while enhancing efferocytosis of apoptotic cells. RvD1 and protectins act via ALX/FPR2 receptors, promoting a phenotypic switch of macrophages from M1 (pro-inflammatory) to M2 (pro-resolving) [148,149]. In preclinical models of periodontitis, topical RvE1 restored periodontal attachment levels and reduced osteoclastogenesis through downregulation of RANKL and NF-κB pathways, demonstrating direct bone-protective effects [150].

#### 4.4.2. Monoclonal Antibodies (mAbs)

Monoclonal antibodies (mAbs) offer potential for treating periodontal inflammation by targeting specific pro-inflammatory cytokines like IL-1β and TNF-α [151]. While not yet approved for periodontal use, these biologics have shown success in reducing alveolar bone loss and inflammatory infiltrates in experimental periodontitis models and are widely used in other inflammatory diseases like rheumatoid arthritis and Crohn’s disease [152,153]. They work by neutralizing these central cytokines, which are involved in driving bone resorption and tissue destruction [151] (Figure 10).

#### 4.4.3. Epigenetic Modulators

Epigenetic modulators are agents being explored to treat periodontal disease by reversing aberrant gene expression, which is increasingly linked to the disease’s development [154]. Specifically, Histone deacetylase (HDAC) inhibitors, such as trichostatin A and valproic acid, increase the expression of protective genes like IL-10 while decreasing pro-inflammatory genes, shifting the tissue’s immune response towards tolerance and repair [155]. DNA Methyltransferase (DNMT) inhibitors are being explored for their potential to reactivate genes involved in tissue repair by reversing aberrant DNA hypermethylation, which can silence important genes. By blocking DNMT enzymes, these inhibitors allow for the re-establishment of normal gene expression, including genes crucial for processes like DNA repair, cell cycle regulation, and immune responses that contribute to tissue restoration [156] (Figure 10).

#### 4.4.4. Matrix Metalloproteinase (MMP) Inhibitors

Beyond doxycycline at sub-antimicrobial doses, newer generations of MMP inhibitors are being engineered for selectivity. Broad MMP inhibition risks impairing physiological remodeling, but targeted inhibition of MMP-8 (neutrophil collagenase) and MMP-9 (gelatinase B) can directly reduce connective tissue degradation. Small-molecule inhibitors and monoclonal antibodies against active MMP domains are under development, with early animal studies showing significant reduction in collagen breakdown [157].

#### 4.4.5. Cytokine and Chemokine Modulators

Additional strategies include blockade of chemokine receptors such as CCR2 and CXCR4, which regulate monocyte and T-cell trafficking into periodontal lesions. Antagonists of CCR2 reduce macrophage infiltration and inflammatory burden, while CXCR4 inhibitors modulate neutrophil retention, reducing collateral tissue injury [158].

Host modulation is especially relevant in periodontitis because tissue destruction is driven by an exaggerated host response as much as by microbial challenge. Nevertheless, the closer these interventions move toward systemic immune manipulation, the greater the need for careful safety profiling, selective targeting, and patient stratification, particularly to avoid unwanted suppression of protective immune functions.

### 4.5. Probiotics and Postbiotics

**Figure 11:**
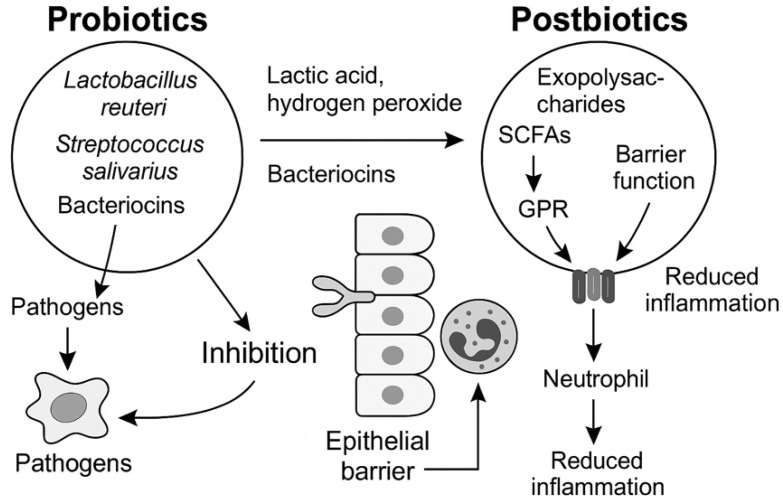
Mechanisms of probiotics and postbiotics in periodontal therapy. Probiotics such as *Lactobacillus reuteri* and *Streptococcus salivarius* inhibit pathogens through competitive adhesion, secretion of bacteriocins, lactic acid, and hydrogen peroxide, while modulating host immunity. Postbiotics, including short-chain fatty acids (SCFAs) and exopolysaccharides, act via G-protein–coupled receptors (GPRs) to enhance epithelial barrier integrity and suppress inflammation, collectively reducing pathogenic load and periodontal tissue destruction. Figure created with Biorender.com.

Restoring microbial homeostasis represents a rational therapeutic goal in periodontitis, which is increasingly recognized as a dysbiosis-driven condition rather than a simple infection. Probiotics and postbiotics offer biologically nuanced approaches by shifting the ecological balance of the oral microbiome and enhancing mucosal immunity without the drawbacks of conventional antibiotics [8,159] (Figure 11).

#### 4.5.1. Mechanisms of Probiotic Action

Probiotics are live microorganisms that confer health benefits when administered in adequate amounts [160]. In the periodontal context, beneficial strains act through several pathways:

1. Competitive Exclusion of Pathogens
  *Lactobacillus reuteri* adheres to epithelial surfaces, blocking colonization sites for *P. gingivalis* and *T. forsythia*. This strain secretes reuterin, a broad-spectrum antimicrobial aldehyde produced from glycerol, which disrupts DNA replication and protein synthesis in red-complex bacteria. *Streptococcus salivarius* secretes bacteriocins (lantibiotics such as salivaricin A and B) that selectively inhibit Gram-negative anaerobes without harming commensals [161].
2. Metabolic Environment Modification
  Probiotic metabolism produces lactic acid and hydrogen peroxide, which acidify the microenvironment and inhibit proteolytic pathogens. Additionally, probiotic-generated biosurfactants interfere with adhesion of pathogens to epithelial cells and hydroxyapatite [162].
3. Immunomodulation
  Certain lactobacilli upregulate IL-10 and TGF-β production via dendritic cell modulation, promoting a tolerogenic immune phenotype. They also suppress pro-inflammatory cytokines such as TNF-α and IL-6 by modulating NF-κB signaling in gingival epithelial cells [163]. In experimental periodontitis models, probiotics have been shown to reduce neutrophil infiltration and downregulate RANKL expression, limiting osteoclastogenesis and alveolar bone resorption [164].

#### 4.5.2. Postbiotics: Functional Metabolites Without Viability Concerns

Postbiotics, defined as non-viable bacterial products or metabolic byproducts, offer a promising alternative to probiotics by overcoming limitations related to viability, storage, and colonization in the oral cavity [ [165]. Among them, short-chain fatty acids (SCFAs) such as butyrate, acetate, and propionate act through G-protein–coupled receptors (GPR41, GPR43, and GPR109A) on gingival epithelial and immune cells, where they enhance epithelial barrier integrity by upregulating tight junction proteins like occludin and claudins, while simultaneously reducing inflammation by suppressing NF-κB signaling and promoting IL-10 secretion [166]. Other important postbiotic molecules include bacteriocins and peptidoglycan-derived fragments; bacteriocins isolated from S. salivarius and Lactobacillus spp. exert direct antimicrobial effects against *P. gingivalis* by disrupting membrane potential, whereas peptidoglycan fragments engage NOD-like receptors to modulate innate immune pathways toward tolerance [167]. Furthermore, exopolysaccharides (EPS) from probiotic strains function as prebiotic substrates for commensals and attenuate macrophage-derived inflammatory cytokine release, while heat-killed Lactobacillus plantarum cell wall components continue to exert anti-inflammatory effects, confirming that postbiotic benefits are independent of bacterial viability [168].

#### 4.5.3. Therapeutic Implications

Clinical trials suggest that adjunctive probiotic therapy, particularly lozenges containing *L. reuteri* or *S. salivarius*, improves bleeding on probing and reduces counts of red-complex bacteria when combined with scaling and root planing [169]. Postbiotics, though less studied clinically, offer advantages in stability and safety, especially in immunocompromised patients where live bacteria may pose risks [170] (Table 3).

Although probiotics and postbiotics are attractive because they support ecological restoration and may carry fewer risks of resistance amplification, their therapeutic effects in periodontology are generally more modest than those expected from targeted anti-biofilm or host-modulating interventions. Their clinical utility may therefore be greatest as adjunctive ecological stabilizers rather than as primary stand-alone therapies.

### 4.6. Pharmacogenomics in Periodontology

**Figure 12:**
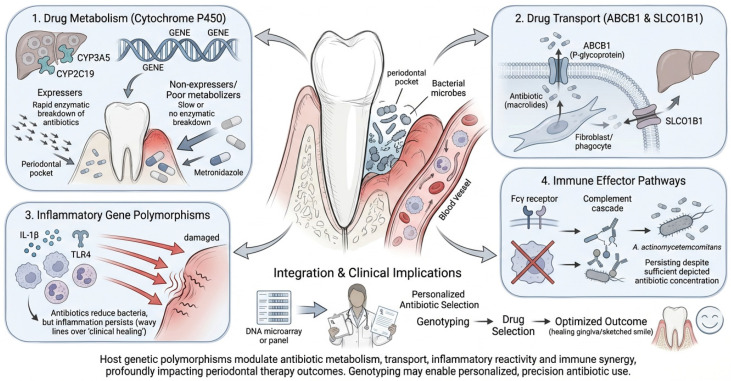
Pharmacogenomic modulation of antibiotic response in periodontitis. Host genetic variability affecting drug metabolism, transport, inflammatory signaling, and immune pathways contributes to differences in antibiotic efficacy and clinical outcomes, supporting the need for personalized periodontal therapy. Arrows indicate the direction of biological processes, transport mechanisms, and clinical effects. Wavy lines represent genetic sequences and polymorphisms influencing gene expression and drug response. Colors are used to differentiate pathological processes, mechanistic pathways, and therapeutic outcomes. Figure created with FigureLab.

While antimicrobial resistance in periodontology has traditionally been viewed as a bacterial phenomenon, host genetic variability exerts equally profound effects on antibiotic outcomes. Pharmacogenomics—the study of how genetic polymorphisms influence drug metabolism, distribution, and therapeutic response—offers critical insights into the observed variability in clinical efficacy and recurrence rates [177] (Figure 12).

#### 4.6.1. Drug Metabolism and Pharmacokinetics

Polymorphisms in cytochrome P450 enzymes significantly modulate systemic antibiotic concentrations. For instance, CYP3A5 expressers demonstrate accelerated clearance of macrolides such as azithromycin, leading to sub-therapeutic drug levels in gingival crevicular fluid and incomplete suppression of periodontal pathogens [60]. Conversely, individuals with non-expressing CYP3A5 alleles retain higher systemic concentrations, potentially enhancing efficacy but also predisposing to side effects. Similarly, CYP2C19 poor metabolizers accumulate metronidazole, whereas ultra-rapid metabolizers fail to achieve therapeutic thresholds, both scenarios undermining predictable outcomes [178].

#### 4.6.2. Drug Transport and Tissue Penetration

Variants in drug transporter genes also affect antibiotic bioavailability within periodontal niches. Polymorphisms in ABCB1 (P-glycoprotein) can limit intracellular accumulation of macrolides in fibroblasts and phagocytes, reducing the reservoir effect that normally provides sustained antibiotic release into periodontal pockets. Alterations in SLCO1B1, a hepatic transporter, further influence systemic clearance and tissue exposure, underscoring the interplay between host genetics and drug distribution [179].

#### 4.6.3. Inflammatory Gene Polymorphisms

Beyond pharmacokinetics, host genetic variability in inflammatory mediators shapes therapeutic responsiveness. IL-1β and TLR4 polymorphisms heighten inflammatory reactivity, driving exaggerated tissue breakdown even under adequate antimicrobial pressure. In such individuals, antibiotics may control bacterial load but fail to prevent continued attachment loss, mimicking microbial resistance [180].

#### 4.6.4. Immune Effector Pathways

Variations in Fcγ receptor and complement cascade genes alter opsonophagocytic efficiency, diminishing the immune system’s capacity to synergize with antibiotics. Reduced clearance of pathogens, particularly *A. actinomycetemcomitans*, perpetuates infection despite pharmacologically sufficient drug concentrations [181].

#### 4.6.5. Clinical Implications

Collectively, these pharmacogenomic determinants highlight why antibiotic therapy in periodontology yields highly variable outcomes across individuals [182]. The clinical observation of drug “resistance” in patients may not always stem from bacterial evolution, but can instead result from a patient’s genetic variations in drug metabolism (e.g., CYP450 enzymes), tissue drug penetration (e.g., ABCB1 transporters), or host immune response (e.g., IL-1 alleles) [183]. Integrating genotyping panels for these genetic factors could enable personalized antibiotic prescriptions by identifying individuals who might be unable to adequately metabolize or absorb a drug, thereby optimizing treatment and mitigating the development of true resistance and broader ecological disruptions, according to the provided statement.

At present, pharmacogenomics should be regarded as a strategic framework for future personalization rather than an immediately deployable periodontal intervention. Its long-term importance is substantial, but broader clinical application will require validation of predictive biomarkers, cost-effective genotyping workflows, and integration into decision-making algorithms that are practical for dental settings.

### 4.7. In Vivo Evidence and Clinical Translation of Emerging Therapeutic Strategies

Despite substantial advances in mechanistic understanding, the translation of emerging therapeutic strategies for periodontal infections from in vitro systems to in vivo and clinical settings remains heterogeneous. The extent of clinical validation varies considerably across modalities, reflecting differences in biological complexity, delivery feasibility, and regulatory readiness.

Antimicrobial peptides (AMPs) have demonstrated promising efficacy in animal models of periodontitis, where topical or locally delivered formulations have been associated with reductions in bacterial load, attenuation of inflammatory infiltrates, and preservation of periodontal attachment [184]. Experimental studies involving peptides such as LL-37 and synthetic analogs have shown not only antimicrobial activity against key periodontal pathogens but also enhancement of wound healing and angiogenesis [185,186]. However, clinical evidence remains limited, with only early-phase trials evaluating peptide-based formulations such as pexiganan in localized delivery systems. Challenges related to peptide stability, proteolytic degradation in the oral environment, and formulation constraints continue to hinder widespread clinical application.

Quorum sensing inhibitors (QSIs) remain largely confined to preclinical investigation. In vitro and animal studies have demonstrated their ability to suppress virulence gene expression, disrupt biofilm maturation, and enhance susceptibility of pathogens to host immune clearance or adjunctive antimicrobial agents [187,188]. Nevertheless, robust in vivo periodontal models are relatively scarce, and well-designed clinical trials evaluating QSIs in periodontal therapy are currently lacking. This gap underscores a critical barrier to clinical translation despite strong mechanistic rationale.

Nanotechnology-based drug delivery systems represent one of the most advanced areas in terms of translational progress. In vivo studies have consistently demonstrated that nanoparticle-encapsulated antimicrobials—such as doxycycline, metronidazole, and chlorhexidine—achieve sustained release, improved penetration into biofilms, and enhanced retention within periodontal pockets. These properties translate into improved clinical parameters, including reductions in probing depth and gains in clinical attachment in experimental and early clinical settings [189,190]. Preliminary clinical studies have reported favorable outcomes with nanoparticle-based formulations compared to conventional local delivery systems; however, long-term safety, cytotoxicity profiles, and regulatory considerations remain under active investigation.

Host modulation therapies (HMT) have achieved partial clinical translation. Sub-antimicrobial dose doxycycline is already an established adjunctive therapy with demonstrated clinical benefits in reducing matrix metalloproteinase activity and slowing connective tissue breakdown. Beyond this, specialized pro-resolving mediators and monoclonal antibodies have shown promising results in animal models, including modulation of inflammatory pathways and reduction of alveolar bone loss. However, their translation into routine periodontal practice is limited by factors such as delivery challenges, cost, and the need for highly selective targeting to avoid systemic immunosuppression [191,192].

Microbiome-based approaches, particularly probiotics, have the most substantial body of clinical evidence among emerging strategies. Multiple randomized controlled trials have demonstrated that adjunctive probiotic therapy—especially formulations containing Lactobacillus reuteri and Streptococcus salivarius—can improve clinical outcomes such as bleeding on probing, pocket depth reduction, and suppression of red-complex pathogens when used alongside scaling and root planing. In contrast, postbiotics remain at an earlier stage of development, with strong mechanistic support but limited direct clinical validation [193,194].

Pharmacogenomics, while not a therapeutic modality per se, is emerging as a critical component of precision periodontal care. Current evidence is largely associative, linking genetic polymorphisms in drug-metabolizing enzymes, transporters, and inflammatory mediators with variability in treatment response. However, prospective clinical studies validating pharmacogenomic-guided treatment strategies in periodontology remain limited, and integration into routine clinical workflows has yet to be established [195,196,197,198].

Overall, the translational landscape of emerging periodontal therapies is uneven. Nanotechnology-based delivery systems and probiotics currently demonstrate the highest levels of clinical applicability, whereas antimicrobial peptides, quorum sensing inhibitors, and pharmacogenomic approaches remain promising but require further validation through well-designed in vivo studies and controlled clinical trials.

## 5. Future Perspectives

- The next generation of drug therapies for bacterial infections in periodontology must transcend the limitations of conventional antibiotics by integrating molecular precision, ecological stability, and systemic health considerations. A central priority is the development of resistance-conscious therapeutics that suppress virulence rather than indiscriminately killing bacteria. Quorum sensing inhibitors (QSIs) and antimicrobial peptides (AMPs) exemplify this paradigm by attenuating biofilm pathogenicity and modulating host responses without imposing strong selective pressure for resistance.
- Nanotechnology offers unique opportunities to overcome pharmacokinetic barriers, enabling targeted delivery of antibiotics, AMPs, or natural bioactives directly into periodontal pockets with controlled release profiles. The incorporation of stimuli-responsive designs, such as pH- or enzyme-triggered release, promises drug activation precisely at disease sites while minimizing off-target effects. Similarly, ligand-functionalized nanoparticles can selectively bind to pathogenic species like *P. gingivalis*, amplifying therapeutic specificity.
- Parallel to antimicrobial innovation, host modulation therapy (HMT) is likely to become central to periodontal pharmacology. The discovery of specialized pro-resolving lipid mediators (SPMs), combined with biologics targeting cytokines and MMPs, signals a shift from immune suppression to immune reprogramming. Epigenetic drugs that rewire inflammatory gene expression may offer long-lasting tissue protection and could be integrated into combination therapies with antimicrobials for synergistic outcomes.
- Equally important is the role of the oral microbiome as a therapeutic target. Probiotics and postbiotics represent ecological interventions designed to restore microbial balance while reinforcing mucosal immunity. Advances in synthetic biology may enable engineered probiotic strains that secrete designer antimicrobials, immunoregulatory cytokines, or biofilm-disrupting enzymes directly within periodontal niches.
- Finally, the future of periodontal pharmacotherapy will be shaped by precision medicine and digital health. Salivary diagnostics and metagenomic sequencing can inform patient-specific microbial and host profiles, guiding tailored drug regimens. Pharmacogenomic testing (e.g., CYP450 variants affecting macrolide metabolism, IL-1β polymorphisms influencing inflammatory susceptibility) may predict responsiveness to both antibiotics and host-modulating agents. Artificial intelligence (AI)-driven drug discovery and treatment optimization, coupled with 3D-printed delivery devices, are poised to revolutionize how therapies are designed and applied in clinical practice.

## 6. Conclusions

The management of bacterial infections in periodontology is undergoing a paradigm shift from reliance on broad-spectrum antibiotics toward more targeted, biologically informed, and precision-oriented therapeutic strategies. While conventional antibiotics remain clinically relevant as adjuncts to mechanical debridement, their long-term effectiveness is increasingly compromised by antimicrobial resistance, biofilm-mediated tolerance, pharmacokinetic limitations, and disruption of the commensal microbiota.

Emerging therapeutic approaches offer distinct advantages by addressing these limitations through diverse mechanisms. Among these, nanotechnology-based drug delivery systems and probiotic interventions currently demonstrate the highest level of clinical applicability, supported by growing in vivo and early clinical evidence showing improved drug retention, biofilm penetration, and favorable clinical outcomes. In contrast, antimicrobial peptides and quorum sensing inhibitors exhibit strong mechanistic potential in targeting bacterial virulence and biofilm dynamics with reduced selective pressure for resistance; however, their clinical translation remains limited by formulation challenges and insufficient validation in human studies. Host modulation therapies represent a critical complementary approach by targeting the dysregulated inflammatory response that drives periodontal tissue destruction, although their broader clinical implementation requires careful optimization to ensure safety and selectivity. Pharmacogenomics, while not a direct therapeutic modality, introduces an essential dimension of personalization by enabling prediction of treatment response based on individual genetic profiles, although its integration into routine clinical practice is still evolving.

Taken together, the current evidence suggests that no single therapeutic strategy is sufficient to address the multifactorial nature of periodontitis. The most promising future direction lies in integrative, combination-based approaches that simultaneously target microbial virulence, biofilm resilience, host inflammatory pathways, and ecological balance. Advancing these strategies will require well-designed longitudinal clinical trials, standardized evaluation frameworks, and translational models that bridge mechanistic insights with clinical applicability. Ultimately, the convergence of targeted therapeutics, precision medicine, and microbiome-aware interventions is expected to redefine periodontal treatment toward more effective, sustainable, and patient-specific care.

## Figures and Tables

**Table 1:** Comparative overview of conventional antibiotics and antimicrobial peptides (AMPs) in periodontology.

Feature	Conventional Antibiotics	Antimicrobial Peptides (AMPs)	References
Primary Mechanism of Action	Target specific bacterial processes: protein synthesis inhibition (tetracyclines, macrolides), DNA damage (metronidazole), or cell wall synthesis inhibition (β-lactams).	Disrupt bacterial membranes via electrostatic binding to phosphatidylglycerol/cardiolipin, pore formation, cytoplasmic leakage; some also bind DNA/RNA.	[71,72]
Host Modulation	Limited: doxycycline inhibits MMPs; azithromycin reduces cytokine release.	Strong: LL-37 and defensins modulate cytokine expression, promote angiogenesis, enhance wound healing, and regulate osteoclastogenesis.	[25,33,73]
Target Specificity	Species- or pathway-specific (e.g., anaerobes for metronidazole, Gram-positive for macrolides).	Broad-spectrum: act on Gram-positive, Gram-negative, fungi, and viruses without reliance on single metabolic pathways.	[74,75]
Biofilm Penetration	Poor: EPS matrix restricts drug diffusion; persister cells evade action.	Superior: small, amphipathic molecules penetrate biofilms; can disrupt quorum sensing indirectly.	[76,77]
Resistance Potential	High: due to plasmid-mediated resistance, efflux pumps, ribosomal protection proteins, β-lactamases.	Low: resistance is rare because target is bacterial membrane integrity, which is difficult to alter without loss of viability.	[78,79]
Pharmacokinetics	Requires systemic or local administration; systemic use limited by plasma binding and toxicity; local delivery short-lived.	Rapid degradation by proteases; stability improved by D-enantiomer AMPs, cyclization, or nanoparticle encapsulation.	[72,80]
Examples in Periodontology	Tetracyclines, metronidazole, amoxicillin, azithromycin, minocycline microspheres.	Endogenous (LL-37, hBD-2, hBD-3); synthetic (pexiganan, novispirin, omiganan); modified (D-enantiomeric AMPs, AMP hydrogels).	[11,81]

**Table 2:** Expanded overview of quorum sensing inhibitors (QSIs) relevant to periodontology, including representative compounds, molecular targets, inhibitory concentrations and periodontal relevance.

Type of QSI	Representative Compound	Molecular Target/Mechanism	Reported Inhibitory Concentration	Evidence Model/Periodontal Relevance	Effects in Periodontitis	References
Synthetic compounds	Furanone derivatives	Structural analogs of AI-2/AHL-type signals; competitively interfere with receptor-level signaling and destabilize QS-controlled transcriptional responses	NR in periodontal source	Mainly in vitro oral-biofilm/QS literature	Reduce *P. gingivalis* virulence signaling, impair biofilm maturation, and may increase susceptibility to adjunctive antimicrobials	[108,112,113]
Synthetic compounds	Halogenated furanones	Interfere with LuxS/AI-2 signaling and downstream transcription; quorum-quenching effect through receptor interference	NR in periodontal source	Preclinical/oral biofilm QS literature	Attenuate virulence-gene expression and weaken established biofilm architecture	[112,114]
Synthetic compounds	Diketopiperazines (DKPs)	Interfere with cyclic peptide signaling and multispecies community communication	NR in periodontal source	Mainly in vitro multispecies biofilm models	Reduce adhesion, motility, and interspecies communication relevant to dysbiotic plaque development	[108,112,115]
Synthetic/nano-enabled compounds	Quantum curcumin (CurQDs)	Curcumin-based nanoformulation with anti-gingipain, antibiofilm, and likely QS-interfering effects through virulence suppression	MIC 1.114 μM against *P. gingivalis* ATCC 33277; MBIC50 0.557 μM; MBIC90 17.826 μM	In vitro, including mixed biofilm model relevant to chronic periodontitis	Strong inhibition of *P. gingivalis* growth, gingipain activity, and mixed-species biofilm biomass	[116,117]
Natural compounds	Quercetin	Flavonoid; suppresses gingipain activity, hemagglutination, hemolysis, biofilm formation, and virulence-gene expression; proposed interference with LuxS-associated virulence regulation	MIC 200 μM; MBC 400 μM against planktonic *P. gingivalis*	In vitro *P. gingivalis* virulence and biofilm model	Inhibits gingipains, reduces biofilm formation at sub-MIC levels, downregulates virulence and iron/heme-utilization genes	[118,119]
Natural compounds	Epigallocatechin gallate (EGCG)	Polyphenol; inhibits biofilm formation, damages bacterial envelope, suppresses virulence traits, and is widely discussed as a quorum-quenching candidate in oral pathogens	MIC 97.5 μg/mL; MBC 187.5 μg/mL against *P. gingivalis*	In vitro plus in vivo mouse periodontitis evidence	Destroys established *P. gingivalis* biofilms, inhibits new biofilm formation, reduces volatile sulfur compound production; oral administration alleviated *P. gingivalis*-associated periodontitis in mice	[119,120,121]
Natural compounds	Curcumin	Suppresses AI-2-associated signaling, gingipain activity, oxidative stress, and biofilm-associated virulence	NR from accessible primary periodontal source in standard curcumin form	In vitro and experimental periodontitis literature	Reduces inflammatory response, inhibits pathogenic biofilm growth, and suppresses key virulence pathways in *P. gingivalis*	[117,122,123]
Natural compounds	Garlic-derived ajoene	Quorum-quenching sulfur compound; disrupts QS-regulated virulence and biofilm coordination in anaerobic pathogens	NR in periodontal source	Primarily preclinical/oral biofilm QS literature	Inhibits mixed-species biofilm formation and is considered a promising adjunctive quorum-quenching agent	[112,124]
Natural compounds	Rosmarinic acid	Plant-derived quorum-quenching candidate reported in oral-biofilm QS reviews	NR in periodontal source	Preclinical/oral biofilm review evidence	Potential attenuation of bacterial communication and plaque-biofilm organization	[112]
Natural compounds	Iberin/organosulfur plant-derived QS inhibitors	Interfere with signal generation or response pathways involved in virulence regulation	NR in periodontal source	Preclinical/oral biofilm review evidence	Considered potential quorum quenchers against periodontal bacteria and related oral biofilms	[112]

**Table 3:** Comparative overview of probiotics and postbiotics in periodontal therapy.

Category	Representative Agents	Molecular Mechanisms	Therapeutic Outcomes in Periodontitis	References
Probiotics	*Lactobacillus reuteri*	Produces reuterin (disrupts DNA replication/protein synthesis); lactic acid and H_2_O_2_ lower pH, inhibiting proteolytic pathogens	Inhibits red-complex bacteria (*P. gingivalis*, *T. forsythia*), reduces bleeding on probing, lowers pocket depth [164]	[171]
	*Streptococcus salivarius*	Secretes bacteriocins (salivaricins A, B) disrupting Gram-negative anaerobe membranes	Suppresses pathogen overgrowth, promotes colonization by commensals	[171]
	Various *Lactobacillus* spp.	Stimulate IL-10, TGF-β, suppress TNF-α, IL-6 via NF-κB modulation	Reduced inflammation, decreased RANKL-mediated osteoclastogenesis [165]	[172]
Postbiotics	Short-chain fatty acids (butyrate, acetate, propionate)	Bind GPR41, GPR43, GPR109A on epithelial/immune cells; ↑ tight junction proteins, ↓ NF-κB signaling	Enhanced barrier integrity, reduced neutrophil-driven tissue damage [166]	[173]
	Bacteriocins (purified)	Directly disrupt bacterial membrane potential	Pathogen suppression without viability concerns [167]	[174]
	Exopolysaccharides (EPS)	Prebiotic substrates for commensals; modulate macrophage cytokine release	Promote microbial homeostasis, dampen inflammation [168]	[175]
	Heat-killed probiotic cell wall fractions	Engage NOD-like receptors to modulate innate immune response	Viability-independent immunomodulation, safer in immunocompromised patients [169]	[176]

## Data Availability

No new data were created or analyzed in this study.
